# Non-myeloablative allogeneic hematopoietic cell transplantation following fludarabine plus 2 Gy TBI or ATG plus 8 Gy TLI: a phase II randomized study from the Belgian Hematological Society

**DOI:** 10.1186/s13045-014-0098-9

**Published:** 2015-02-06

**Authors:** Frédéric Baron, Pierre Zachée, Johan Maertens, Tessa Kerre, Aurélie Ory, Laurence Seidel, Carlos Graux, Philippe Lewalle, Michel Van Gelder, Koen Theunissen, Evelyne Willems, Marie-Paule Emonds, Ann De Becker, Yves Beguin

**Affiliations:** Department of Hematology, University of Liège, and CHU of Liège, Sart-Tilman, 4000 Liège, Belgium; ZNA Stuivenberg, Antwerpen, Belgium; AZ Gasthuisberg Leuven, Leuven, Belgium; Ghent University Hospital, Ghent, Belgium; Department of Statistics, University of Liège, and CHU of Liège, Liège, Belgium; Mont-Godine University Hospital (UCL), Yvoir, Belgium; Jules Bordet Institute (ULB), Bruxelles, Belgium; Maastricht University Medical Center, Maastricht, The Netherlands; Jessa Ziekenhuis, Hasselt, Belgium; HLA Red Cross Flanders, Mechelen, Belgium; Universitair Ziekenhuis Brussel (UZ Brussels), Brussels, Belgium

**Keywords:** allo-HCT, Non-myeloablative conditioning, TBI, TLI, ATG, GVHD, Graft-versus-leukemia effects

## Abstract

**Background:**

Few studies thus far have compared head-to-head different non-myelooablative conditioning regimens for allogeneic hematopoietic cell transplantation (allo-HCT).

**Methods:**

Here, we report the results of a phase II multicenter randomized study comparing non-myeloablative allo-HCT from HLA-identical siblings (n = 54) or from 10/10 HLA-matched unrelated donors (n = 40) with either fludarabine plus 2 Gy total body irradiation (Flu-TBI arm; n = 49) or 8 Gy TLI + anti-thymocyte globulin (TLI-ATG arm; n = 45) conditioning.

**Results:**

The 180-day cumulative incidences of grade II-IV acute GVHD (primary endpoint) were 12.2% versus 8.9% in Flu-TBI and TLI-ATG patients, respectively (P = 0.5). Two-year cumulative incidences of moderate/severe chronic GVHD were 40.8% versus 17.8% in Flu-TBI and TLI-ATG patients, respectively (P = 0.017). Five Flu-TBI patients and 10 TLI-ATG patients received pre-emptive DLI for low donor chimerism levels, while 1 Flu-TBI patient and 5 TLI-ATG patients (including 2 patients given prior pre-emptive DLIs) received a second HCT for poor graft function, graft rejection, or disease progression. Four-year cumulative incidences of relapse/progression were 22% and 50% in Flu-TBI and TLI-ATG patients, respectively (P = 0.017). Four-year cumulative incidences of nonrelapse mortality were 24% and 13% in Flu-TBI and TLI-ATG patients, respectively (P = 0.5). Finally, 4-year overall (OS) and progression-free survivals (PFS) were 53% and 54%, respectively, in the Flu-TBI arm, versus 54% (P = 0.9) and 37% (P = 0.12), respectively, in the TLI-ATG arm.

**Conclusions:**

In comparison to patients included in the Flu-TBI arm, patients included in the TLI-ATG arm had lower incidence of chronic GVHD, higher incidence of relapse and similar OS.

**Trial registration:**

The study was registered on ClinicalTrial.gov (NCT00603954) and EUDRACT (2010-024297-19).

**Electronic supplementary material:**

The online version of this article (doi:10.1186/s13045-014-0098-9) contains supplementary material, which is available to authorized users.

## Background

Allogeneic hematopoietic cell transplantation (allo-HCT) following non-myeloablative conditioning is increasingly used as treatment for hematological malignancies in older patients or those with medical comorbidities [1,2]. This approach relies mostly on immune-mediated graft-versus-tumor effects for tumor eradication [3,4]. One of the most widely used non-myeloablative conditioning associates fludarabine (90 mg/m^2^ total dose) and 2 Gy total body irradiation (TBI) [3-10]. This regimen can be safely performed in an outpatient setting but is associated with a relatively high incidence of graft-versus-host disease (GVHD) [3-7].

In an effort to prevent GVHD, the Stanford group has developed another non-myeloablative conditioning that combines total lymphoid irradiation (TLI, 8 Gy total dose) with ATG (7.5 mg/kg Thymoglobulin® total dose) [11-15]. This approach allowed sustained engraftment with a low incidence of GVHD through polarization of donor T cells by recipient iNKT cells (still present at transplantation because they are relatively resistant to the conditioning) towards a Th2 phenotype [16].

As these two promising regimens had never been compared head-to-head, the Belgian Hematological Society (BHS)-transplantation committee initiated a phase II multicenter randomized study comparing non-myeloablative allo-HCT with either fludarabine plus 2 Gy TBI (Flu-TBI arm) or 8 Gy TLI + ATG (TLI-ATG arm) conditioning.

## Results

### Patients

One hundred and seven patients were randomized in the Flu-TBI (n = 55) or TLI-ATG (n = 52) arms between January 2008 and March 2011 in one of the 9 participating centers (Additional file 1: Table S1). Thirteen patients (6 in the Flu-TBI and 7 in the TLI-ATG arm) were excluded from analysis because they did not meet the inclusion criteria at the time of the start of the conditioning (disease relapse before the start of the conditioning (n = 5), ineligible for further irradiation (n = 3), donor refusal to give peripheral blood stem cells (PBSC) (n = 2), HLA-mismatched donor (n = 2), and poor PS precluding transplantation (n = 1)). One patient randomized in the Flu-TBI arm received the TLI-ATG conditioning (and was analyzed in intention to treat in the Flu-TBI arm). Thus, the analysis includes data from 94 patients randomized to the Flu-TBI (n = 49) or TLI-ATG (n = 45) arm (Table 1). The 2 groups were well balanced with the exception of higher numbers of transplanted CD34^+^ cells (P = 0.02) and a suggestion for a higher proportion of patient with high disease risk according to the Kahl *et al.* classification [17] (P = 0.17) in the TLI-ATG arm. Results were analyzed as of 3 October 2013. Median follow-up for surviving patients was 45 (range, 19–65) months.

**Table 1:** **Patient characteristics**

	**Flu–TBI**	**TLI-ATG**	**p value**
**Number of patients**	49	45	
**Patient age, Median (range), years**	60 (38–73)	59 (32–71)	0.2
**Patient gender (male/female), # of patients**	35/14	29/16	0.5
**Donor type; # of patients**			
HLA-identical sibling/10/10 HLA-allele matched URD	29/20	25/20	0.8
**Donor age, Median (range), years**	51 (19–66)	46 (19–69)	0.6
**Female donor to male recipient, # of patients**	12	9	0.6
**CMV-serostatus (donor/patient), # of patients**			0.3
−/−	12	13^†^	
−/+	17	12	
+/−	8	3	
+/+	11*	16	
**Disease at transplantation; # of patients**			
Acute myeloid leukemia	17	16	
Acute lymphoblastic leukemia	4	1	
Chronic lymphocytic leukemia	7	3	
Myelodysplatic syndrome/	9	8	
Chronic myelomonocytic leukemia	2	3	
Multiple myeloma	3	2	
Myeloproliferative disorder	2	1	
Non-Hodgkin lymphoma	5	10	
Waldenström disease	0	1	
**Disease risk** [17]**: low/standard/high; # of patients**	15/24/10	12/18/15	0.4^‡^
**Comorbidity (HCT-CI score)** [18]**; median (range)**	1 (0–8)	0 (0–6)	0.2
**Performance status: 0/1/2; # of patients**	17/30/2	22/21/2	0.4
**Prior autologous HCT; # of patients**	10	6	0.4
**Graft composition (× 10** ^**6**^ **/kg recipient)**			
CD34; median (range)	5.6 (2.1–11.5)	6.6 (2.4–11.8)	0.02
CD3; median (range)	334 (76–647)	291 (73–834)	0.8

*the CMV serostatus of one recipient (CMV sero-positive donor) is missing; ^†^the CMV serostatus of one recipient (CMV sero-negative donor) is missing; ^‡^P = 0.17 when only the % of patients with high-risk disease was compared.

### Protocol deviation

In the Flu-TBI arm, major protocol deviations included the use of cyclosporine instead of tacrolimus for GVHD prophylaxis (n = 5), incorrect administration of fludarabine (4 days instead of 3 days (n = 1) or incorrect schedule of fudarabine or TBI administration (n = 4; including 2 of the 4 patients given cyclosporine for GVHD prophylaxis), or administration of the TLI-ATG conditioning instead of the Flu-TBI regimen (n = 1). In the TLI-ATG arm, major protocol deviations occurred in 9 patients and included incorrect timing of ATG administration (n = 5; too close to transplantation with the last ATG dose on day −6 (n = 1), −5 (n = 2), or–3 (n = 2)), incorrect dose of ATG (n = 3; 10 mg/kg total dose (n = 2), 3 mg/kg total dose (n = 1)), cyclosporine instead of tacrolimus (n = 2), and 8 Gy TLI administered in 9 fractions instead of 10 (n = 1).

### Engraftment

**Figure 1:**
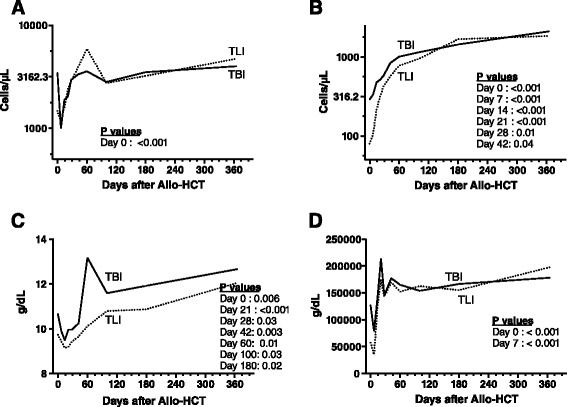
**Hematologic recovery in the 2 groups the first year after transplantation. A)** Neutrophil count (ANC), **B)** lymphocyte count (ALC), **C)** hemoglobin (Hb) levels and **D)** platelet levels.

In comparison to Flu-TBI patients, TLI-ATG patients had significantly lower absolute neutrophil counts on day 0, lower absolute lymphocyte counts from day 0 to day 42, lower platelet counts on days 0 and 7, and lower hemoglobin levels on day 0 and from day 21 to day 180 after transplantation (Figure 1). Accordingly, 30 of 49 Flu-TBI patients versus 38 of 45 TLI-ATG patients were given at least 1 red blood cell transfusion the first 100 days after transplantation (P = 0.02), while 14 of 49 Flu-TBI patients versus 26 of 45 TLI-ATG patients were given at least 1 platelet transfusion during that period (P = 0.006).

**Figure 2:**
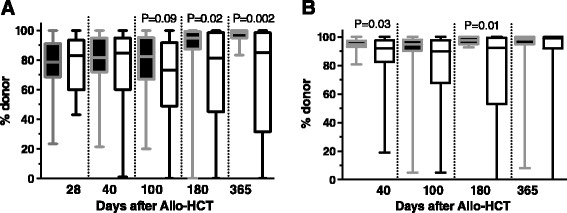
**Chimerism levels in Flu-TBI (black boxes) and TLI-ATG (white boxes) patients. A)** T cell chimerism levels, and **B)** Bone marrow (BM) chimerism levels.

Donor T-cell chimerism levels were lower in the TLI-ATG arm on days 180 and 365 after HCT, while TLI-ATG patients had also lower marrow chimerism levels on days 40 and 180 after allo-HCT (Figure 2). Three patients in the Flu-TBI arm and 4 patients in the TLI-ATG arm had graft rejection (defined as ≤ 5% donor chimerism in T cells, total white blood cells and/or total bone marrow cells). Further, 5 Flu-TBI patients and 10 TLI-ATG patients received pre-emptive DLI for low donor chimerism levels (another TLI-ATG recipient received pre-emptive DLI for relapse prevention), while 1 Flu-TBI patient and 5 TLI-ATG patients (including 2 given prior pre-emptive DLIs) received a second HCT for poor graft function, graft rejection, or disease progression (P = 0.1).

### GVHD

**Figure 3:**
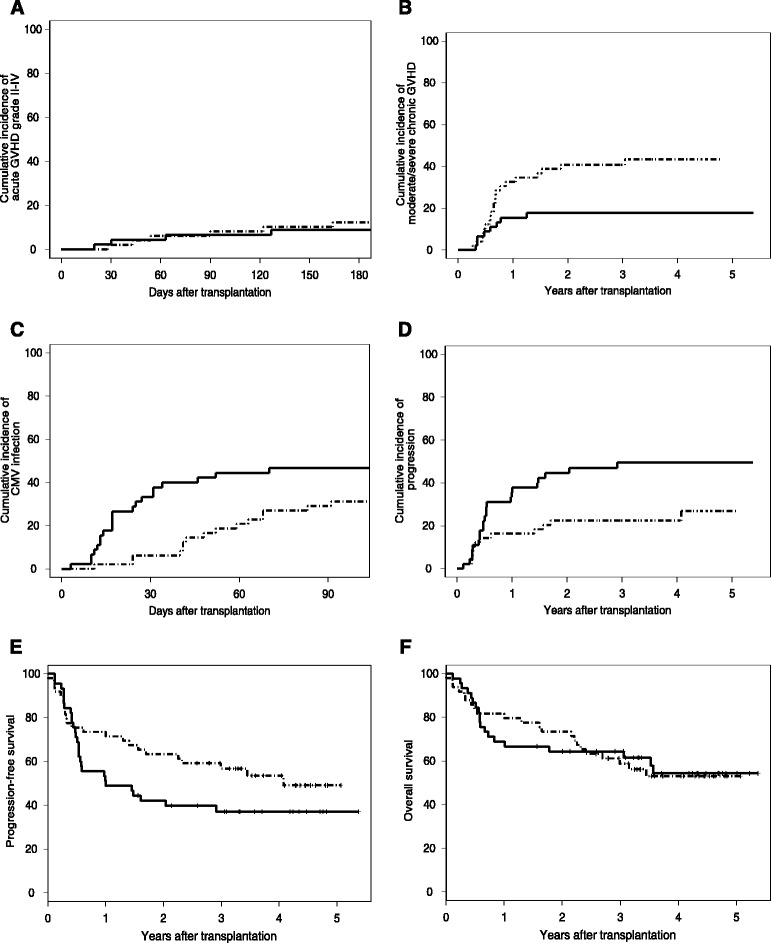
**Transplantation outcomes. A)** 180-day cumulative incidence of grade II-IV acute GVHD. **B)** 5-year cumulative incidence of moderate/severe chronic GVHD. **C)** 100-day cumulative incidence of CMV reactivation. **D)** 5-year cumulative incidence of progression. **E)** 5-year progression-free survival. **F)** 5-year overall survival. Broken line: fludarabine (90 mg/m^2^) plus 2 Gy total body irradiation; continuous line = 8 Gy total lymphoid irradiation plus ATG thymoglobulin^R^ (7.5 mg/kg).

Before day 180, 6/49 Flu-TBI patients had grade II (n = 4), grade III (n = 1) or grade IV (n = 1) acute GVHD while 4/45 TLI-ATG patients had grade II (n = 3) or IV (n = 1, occurring after pre-emptive DLI for poor chimerism) acute GVHD. The 180-day cumulative incidences of grade II-IV acute GVHD were 12.2% versus 8.9% in Flu-TBI and TLI-ATG patients, respectively (P = 0.508) (Figure 3A).

Two-year cumulative incidences of moderate/severe chronic GVHD were 40.8% versus 17.8% in Flu-TBI and TLI-ATG patients, respectively (P = 0.0165) (Figure 3B). In multivariate analysis, TLI-ATG conditioning (HR = 0.3, 95% confidence interval (CI): 0.1-0.8, P = 0.010), and transplantation from a HLA-identical sibling donor (HR = 0.5, 95% CI: 0.2-1.0; P = 0.0495) were associated with a lower incidence of moderate/severe chronic GVHD, while female donor to male recipient was associated with a higher incidence of moderate/severe chronic GVHD (HR 3.8, 95% CI: 1.7-8.5, P = 0.001).

### Infections

Nineteen of 49 Flu-TBI patients (39%) versus 25 of 45 TLI-ATG patients (56%) had a least one episode of bacterial infection the first 100 days after transplantation (P = 0.15). For fungal infections, the figures were 3 of 45 (6%) and 7 of 45 (16%), respectively (P = 0.19). Finally, among CMV-seropositive patients and/or donors, the 100-day cumulative incidence of CMV reactivation was 31% in Flu-TBI patients versus 47% in TLI-ATG patients (P = 0.12; Figure 3C).

### Relapse, nonrelapse mortality and survival

Four-year cumulative incidences of relapse/progression were 22% and 50% in Flu-TBI and TLI-ATG patients, respectively (P = 0.017; Figure 3D). The difference remained statistically significant in multivariate analysis (HR = 2.3, 95% CI 1.1-4.7, P = 0.02). Four-year cumulative incidences of NRM were 24% and 13% in Flu-TBI and TLI-ATG patients, respectively (P = 0.5).

Four-year OS and PFS were 53% and 54%, respectively, in the Flu-TBI arm, versus 54% (P = 0.9) and 37% (P = 0.12), respectively, in the TLI-ATG arm (Figure 3 E-F). In multivariate analyses, there was a trend for lower PFS in patients transplanted for high-risk disease (HR = 2.0, 95% CI: 1.0-4.1, P = 0.07), while higher HCT-CI scores [18] predicted for lower OS (HR = 1.2, 95% CI: 1.0-1.4, P = 0.02). Causes of death are listed in the Table 2.

**Table 2:** **Causes of death**

	**Flu-TBI (n = 22/49)**	**TBI-ATG (n = 19/45)**
Relapse/progression*	10	13
Infection	5	3
Acute GVHD	1	2^†^
Chronic GVHD	2	0
Acute respiratory distress syndrome	1	0
Alveolar hemorrhage	1	0
Epilepsy	1	0
Second malignancy	1	0
Hemolytic anemia	0	1

*defined as every death occurring after relapse/progression; ^†^including one patient after pre-emptive DLI for poor chimerism.

OS and PFS were further recalculated as of December 2014. With a median follow-up for surviving patients of 58.5 months, 5-year OS and PFS were 53% and 50% respectively in Flu-TBI patients, versus 55% (P = 0.96) and 37% (P = 0.14), respectively, in TLI-ATG patients.

## Discussion

This multicenter randomized phase II study compared two non-myeloablative conditioning regimens. Main observations were that although the incidence of grade II-IV acute GVHD was comparable in both arms, the TLI-ATG regimen was associated with a significantly lower incidence of moderate/severe chronic GVHD. The similar incidence of acute GVHD in the 2 arms was due to a lower than anticipated incidence of grade II-IV acute GVHD in the TBI arm, perhaps due to a relatively high dose of MMF used in sibling recipients, and to the relatively high targeted tacrolimus levels the first 100 days after transplantation [19]. In contrast, the low incidence of moderate/severe chronic GVHD in TLI-ATG patients is consistent with prior publications from the Stanford group [11,12], while the 40% incidence of chronic GVHD in Flu-TBI patients is also in agreement with observation from the Seattle consortium [8,10]. Although previous studies have demonstrated a lower incidence of chronic GVHD in patients given ATG [20-22], the low incidence of chronic GVHD observed in current TLI-ATG recipients is unlikely due to ATG only, given that other studies have demonstrated that median serum active ATG levels the day of transplantation after TLI-ATG regimen are < 5 mg/L [11,23], well below the threshold associated with lower incidence of chronic in a recent study by Chawla *et al.* (8.12 mg/L) [24].

The TLI-ATG regimen was also associated with a higher incidence of relapse, although these results should be taken with caution given the heterogeneity of diagnoses and status at transplantation in our study. Nevertheless, supporting our data, the incidence of relapse in TLI-ATG patients in the current study (50% at 4 years) is comparable to what has been observed by the Stanford group (53% at 4-year) in a larger cohort of patients [12]. This observation is also in accordance with prior studies that observed higher risks of relapse with lower donor T-cell chimerism [3,12,25,26] and absence of chronic GVHD [3,4,27,28]. This higher incidence of relapse in TLI-ATG patients translated into a trend for lower PFS, but, importantly, OS was identical in the 2 arms.

## Conclusions

In summary, in comparison to patients included in the Flu-TBI arm, patients included in the TLI-ATG arm had lower incidence of chronic GVHD, higher incidence of relapse, and similar OS. Current efforts are focusing at decreasing the incidence of GVHD without affecting the relapse incidence in Flu-TBI patients (ClinicalTrial.gov # NCT01231412 & NCT01428973), and at preventing relapse in patients with low donor chimerism levels early after transplantation in TLI-ATG patients (ClinicalTrial.gov # NCT01392989).

## Methods and study design

### Conditioning regimen and transplant procedure

In the Flu-TBI arm, conditioning consisted of fludarabine 30 mg/m^2^ on days −4, −3 and −2, followed by a singe dose of 2 Gy TBI administered on day 0 (TBI administration on day −1 was also allowed). In the TLI-ATG arm, conditioning consisted of 8 Gy TLI (80 cGy daily, starting 11 days before transplantation, until a total of 10 doses (8 Gy) had been delivered) and ATG (Thymoglobulin®, Genzyme) given i.v. at a dose of 1.5 mg/kg/d from days −11 through −7. In both regimens postgrafting immunosuppression included mycophenolate mofetil (MMF) administered orally from the evening of day 0 through day 28 (HLA-identical sibling donors) or day 42 (10/10 HLA allele matched unrelated donors) at a dose of 15 mg/kg t.i.d., and tacrolimus administered orally from day −3. Tacrolimus doses were adapted to achieve whole blood through levels between 15 and 20 ng/ml the first 28 days and between 10–15 ng/ml thereafter. Full doses were given until day 100 (sibling recipients) or 180 (unrelated recipients). Doses were then progressively tapered to be discontinued (in the absence of GVHD) by day 180 (sibling donors) or 365 (unrelated donors). Acute GVHD and late acute GVHD were graded using international criteria, while chronic GVHD was graded according to the NIH criteria [29].

### Study design

The study planned the inclusion of 100 patients. The study was approved by the Ethics Committee of the University of Liège (central ethic committee) and by the ethics committee of the participating centers. The study was registered on ClinicalTrial.gov (NCT00603954; https://www.clinicaltrial.gov/ct2/show/NCT00603954?term=NCT00603954&rank=1) and EUDRACT (2010-024297-19; https://www.clinicaltrialsregister.eu/ctr-search/trial/2010-024297-19/BE).

### Inclusion and exclusion criteria

Inclusion criteria were: 1) Hematological malignancies confirmed histologically and not rapidly progressing; 2) Theoretical indication for a myeloablative allo-HCT, but not feasible because of age, patient refusal, or comorbidity, or planned tandem autologous/allogeneic HCT; 3) age ≤ 75 years of age; 4) HLA-identical sibling donor or 10/10 HLA –A, −B, −C, −DRB1, −DQB1 allelic matched related or unrelated donor fit to (and willing to) give G-CSF mobilized peripheral blood stem cells (PBSC), and 5) Signed informed written consent. Main exclusion criteria included: 1) HIV positivity; 2) Non-hematological malignancy(ies) (except non-melanoma skin cancer) < 3 years before allo-HCT; 3) Life expectancy severely limited by disease other than malignancy; 4) Administration of cytotoxic agent(s) for “cytoreduction” within three weeks prior to initiating the non-myeloablative transplant conditioning (Exceptions were hydroxyurea and imatinib mesylate); 5) CNS involvement with disease refractory to intrathecal chemotherapy; 6) Terminal organ failure, except for renal failure; 7) Uncontrolled infection; 8) Karnofsky Performance Score <70%; and 9) Previous radiation therapy precluding the use of 2 Gy TBI or 8 Gy TLI.

### Endpoints

The primary endpoint was the 180-day incidence of grade II-IV acute GVHD. Secondary endpoints included: 1) hematopoietic (whole blood and T-cell chimerisms) engraftment and incidence of graft rejection; 2) Incidence of grade I-IV and III-IV acute GVHD; 3) Incidence of chronic GVHD; 4) Quality and timing of immunologic reconstitution (that will be reported in a separate analysis [23]); 5) Incidences of infections; and 6) incidence of relapse (RI), nonrelapse mortality (NRM), progression-free survival (PFS) and overall survival (OS).

### Randomization

Patients were randomized 1/1 according to a randomization list obtained with the Graphpad QuickCalcs software. Randomization was stratified by center. Randomizations were carried out by a data manager or by an investigator from the University of Liège.

### Statistical analyses

Graft composition, hematological recovery and chimerism levels among groups were compared using the Mann–Whitney test. Comparison of the number of patients who developed at least one infectious episode in the 2 groups was performed using the Fisher’s exact test. OS and PFS were estimated by the Kaplan-Meier method. Cumulative incidence curves were used for GVHD, CMV infection, and relapse incidences (RI) with death as a competitive risk, and for nonrelapse mortality (NRM) with relapse as a competitive risk. Patients given a second allogeneic HCT were censured for GVHD analyses. Multivariate Cox models were fit for analyzing acute and chronic GVHD, PFS, OS and RI. Potential factors examined in the Cox models for GVHD included study arm, female donor to male recipient versus other gender combinations, related versus unrelated donor, donor and recipient CMV-serostatus, and patient age. Factors analyzed for OS, PFS, NRM and RI included study arm, female donor to male recipient versus other gender combinations, related versus unrelated donor, patient age, disease risk of relapse (as defined by Kahl et al. [30]), and HCT-CI score [18] (only for NRM, OS and PFS). There were stopping rules (in each group separately) for graft rejection > 15% at day 180, and for NRM > 35% at day 180, while a planned interim analysis was performed after 60 patients. Results were analyzed as of 3 October 2013. Statistical analyses were performed with Prism 6 for Macintosh (Graphpad Software, San Diego, CA) and SAS 9.3 for Windows (SAS Institute, Cary, NC); some graphs were drawn with S-Plus 8.2.

## Additional file

Additional file 1: Table S1. Participating centres.

## Abbreviations

Allo-HCT: Allogeneic hematopoietic cell transplantation
Flu: Fludarabine
TBI: Total body irradiation
TLI: Total lymphoid irradiation
PBSC: Peripheral blood stem cells (PBSC)
ATG: Anti-thymocyte globulin
HLA: Human Leukocyte Antigen
PS: Performance status
MMF: Mycophenolate mofetil
GVHD: Graft-versus-host disease
CMV: Cytomegalovirus
OS: Overall survival
PFS: Progression-free survival
RI: Relapse incidence
NRM: Nonrelapse mortality
HR: Hazard ratio
HCT-CI: Hematopoietic cell transplantation comorbidity index score
