# Supplementary material for: Non-myeloablative allogeneic hematopoietic cell transplantation following fludarabine plus 2 Gy TBI or ATG plus 8 Gy TLI: a phase II randomized study from the Belgian Hematological Society
Source: J Hematol Oncol. 2015 Feb 6;8:4. doi: 10.1186/s13045-014-0098-9 (PMC4332717; doi:10.1186/s13045-014-0098-9)
Supplement: Additional file 1: Table S1. — Participating centres. [file 13045_2014_98_MOESM1_ESM.docx]

**Table S1: participating centres.**

| **Centre** | **# of eligible patients** |
| --- | --- |
| CHU of Liège, Liège, Belgium | 32 |
| ZNA Stuivenberg, Antwerpen, Belgium | 18 |
| AZ Gasthuisberg Leuven, Leuven, Belgium | 15 |
| Ghent University Hospital, Ghent, Belgium | 9 |
| Mont-Godine University Hospital (UCL), Yvoir, Belgium | 6 |
| Jules Bordet Institute (ULB), Bruxelles, Belgium | 6 |
| University Hospital Maastricht, Maastricht, The Netherlands | 5 |
| Universitair Ziekenhuis Brussel (UZ Brussels), Belgium | 3 |
